# COINSTAC: Decentralizing the future of brain imaging analysis

**DOI:** 10.12688/f1000research.12353.1

**Published:** 2017-08-18

**Authors:** Jing Ming, Eric Verner, Anand Sarwate, Ross Kelly, Cory Reed, Torran Kahleck, Rogers Silva, Sandeep Panta, Jessica Turner, Sergey Plis, Vince Calhoun

**Affiliations:** 1 Datalytic Solutions, Albuquerque, NM, 87106, USA; 2 The Mind Research Network, Albuquerque, NM, 87106, USA; 3 Department of Electrical and Computer Engineering, Rutgers University, Piscataway, NJ, 08854, USA; 4 Department of Psychology and Neuroscience Institute, Georgia State University, Atlanta, GA, 30303, USA; 5 Department of Electrical and Computer Engineering, University of New Mexico, Albuquerque, NM, 87131, USA

**Keywords:** Decentralized algorithm, iterative optimization, data sharing, brain imaging, privacy preserving

## Abstract

In the era of Big Data, sharing neuroimaging data across multiple sites has become increasingly important. However, researchers who want to engage in centralized, large-scale data sharing and analysis must often contend with problems such as high database cost, long data transfer time, extensive manual effort, and privacy issues for sensitive data. To remove these barriers to enable easier data sharing and analysis, we introduced a new, decentralized, privacy-enabled infrastructure model for brain imaging data called COINSTAC in 2016. We have continued development of COINSTAC since this model was first introduced. One of the challenges with such a model is adapting the required algorithms to function within a decentralized framework. In this paper, we report on how we are solving this problem, along with our progress on several fronts, including additional decentralized algorithms implementation, user interface enhancement, decentralized regression statistic calculation, and complete pipeline specifications.

## Introduction

Proliferating neuroimaging data present contemporary neuroscientists with both an exciting opportunity and a cumbersome challenge. The advantages of sharing data are clear. Adding datasets to a study increases sample size, making predictions more certain, and increases diversity, allowing differences between groups to be studied. Although there is indeed an abundance of data, there exist multiple barriers to fully leverage such data. Firstly, a significant amount of existing neuroimaging data has been collected without proper provisions for *post hoc* data sharing. Secondly, researchers must negotiate data usage agreements (DUAs) to collaborate and build models using multiple sources of data that can be anonymized and shared. Sharing data via a DUA is advantageous in that all the variables collected can be studied. However, these DUAs may require months to complete, and the effort to obtain them could be ultimately fruitless, as researchers only know the utility of the data after they have obtained and explored it. Thirdly, even if neuroimaging data can be shared in an anonymized form, the data require a copious amount of storage, and the algorithms applied to the data require significant centralized computational resources. Fourthly, even anonymized data bears a risk of reidentification, especially for subjects who are rare because of a combination of demographic and clinical data. While centralized sharing efforts are powerful and unquestionably should continue, the community needs a family of approaches to address all the existing challenges, including decentralized models that we describe in this paper. One alternative to centralized data sharing is to perform meta-analyses utilizing existing literature to avoid the burden of negotiating DUAs and storing and processing data ( Thompson *et al.*, 2017; Thompson *et al.*, 2014). However, meta-analyses suffer from heterogeneity among studies caused by varying preprocessing methods applied to the data and inconsistent variables collected. In addition, meta-analytic results are not as accurate as those obtained from a centralized analysis.

The Collaborative Informatics and Neuroimaging Suite Toolkit for Anonymous Computation (COINSTAC), proposed by Plis *et al.*, in 2016 ( Plis *et al.*, 2016), solves the abovementioned problems by providing a decentralized platform by which researchers can collaboratively build statistical and machine learning models, while neither transmitting their data nor sacrificing privacy concerns, thanks to differentially private algorithms. COINSTAC can run both meta-analyses and mega-analyses via “single-shot” and “multi-shot” (iterative) computations, respectively. The COINSTAC software (currently in an early prototype) is freely available, open source, and compatible with all major operating systems (Windows, Mac OS, and Linux). It is an easy-to-install, standalone application with a user-friendly, simple, and intuitive interface. By utilizing Docker containers, COINSTAC can run computations in any programming language (including Python, R, Matlab, FORTRAN, and C++) and is easily extensible. We are also building a development community to help users create their own computations, as well.

The use of a decentralized analysis framework has many advantages. For example, decentralized analysis can move beyond meta-analysis via iteration, obtaining a solution equivalent to that of the centralized result. In addition, one can move beyond sharing summary measures—which though plausibly private can still potentially be reidentified—to a more formally private solution. Differential privacy has been touted as a solution to the data sharing and reidentification problem. Developed by Dwork *et al.*, 2006, this approach statistically guarantees privacy and allows for sharing aggregated results without the risk of reidentification ( Dwork *et al.*, 2006).

In the past few years, we have developed many algorithms that run in a decentralized and optionally a differentially private manner. Decentralized computations include ridge regression ( Plis *et al.*, 2016), multi-shot regression ( Plis *et al.*, 2016), independent vector analysis (IVA) ( Wojtalewicz *et al.*, 2017), neural networks ( Lewis *et al.*, 2017), decentralized stochastic neighbor embedding (dSNE) ( Saha *et al.*, 2017), joint independent component analysis (ICA) ( Baker *et al.*, 2015), and two-level differentially private support vector machine (SVM) classification ( Sarwate *et al.*, 2014). To facilitate and accelerate algorithm development, we have created *COINSTAC-simulator*, which allows algorithm developers to prototype and troubleshoot their algorithms before deployment to real consortia in COINSTAC.

Furthermore, we include both input and output functionality to the COINSTAC user interface. For example, the interface for regression can accept data produced by FreeSurfer, with a menu to select the region of interest (ROI) in the brain that will be used as the dependent variable in the statistical analysis. Following the analysis, COINSTAC produces a statistics table for the output of ridge regression, which calculates the global p-values and t-values in a decentralized fashion for each site in the consortium, measuring goodness of fit.

COINSTAC also enables decentralized analyses with multiple computation steps. Easy and flexible computation stacking is a built-in feature in our framework. In this paper, we demonstrate an implementation scheme for specifying and managing multiple computations. With this framework, we can incorporate local computations, such as common preprocessing brain imaging tasks, into the analysis workflow.

A common nuisance among programmers and especially non-expert users is the assembly of an environment to run a computer program. This is a crucial step that may require upgrading an operating system and downloading and installing the latest release of software, a compiler, or a supporting library. Assembly of the environment may involve permission from IT and a substantial amount of troubleshooting, which may lead to a long delay before analysis can begin. Additionally, inconsistent machine state between computers (including operating systems, libraries, and compilers) can lead to inconsistent results from the same computation.

A popular solution to this problem is utilizing a virtual machine (VM) that contains all the dependencies needed to run a program. Because VMs are resource-intensive, many developers have switched to using containers, which are an efficient, lightweight solution to the problem of heterogeneous development environments. Containers only bundle in the supporting software needed to run the program and do not require running a full VM with its own operating system. This reduces the required amount of memory and number of CPUs.

COINSTAC encapsulates individual computations inside Docker containers ( https://www.docker.com/what-docker), which are run in series in a pipeline. Containers holding computations can be downloaded and run locally, which removes the need to assemble a development environment and thus greatly reduces the time to analyze results. This solution will also allow consortium participants to run coordinated preprocessing operations that must often occur before a statistical analysis, such as FreeSurfer processing or voxel-based morphometry. We have already created a Docker container with a standalone SPM package utilizing the Matlab Compiler Runtime. The normalization and coordination of preprocessing operations reduce heterogeneity in the data, creating a solid basis for the main analyses.

## Methods and use cases

### Algorithms for decentralized data analysis

In our previous paper ( Plis *et al.*, 2016), we demonstrated the use of decentralized gradient descent in the optimization of a basic ridge regression model. This decentralized iterative optimization process represents an analysis of virtual data pooling. The resulting model generated in this manner is equivalent to the model generated in centralized repository analysis (i.e., the meta-analysis becomes a *mega-analysis*).

In this paper, we apply the decentralized gradient descent methods to other more advanced algorithms in the neuroimaging domain, including t-distributed nonlinear embedding (tSNE), shallow and deep neural networks, joint ICA, and IVA. These methods are already widely used in the neuroimaging domain, but have not previously been extended to work in a decentralized framework. We demonstrate how these methods can be computed within a decentralized framework and report the algorithm performance compared to a centralized analysis.

***Decentralized tSNE (dSNE).*** A common method of visualizing a dataset consisting of multiple high-dimensional data points is embedding the points into a 2- or 3-dimensional space. Such an embedding serves as an intuitive exploratory tool for quick detection of underlying structure of a dataset. In 2008, van der Maaten and Hinton proposed a method named tSNE to efficiently handle this situation ( Maaten & Hinton 2008). The embeddings produced by tSNE are usually intuitively appealing and interpretable, which makes this method an attractive tool in many domains, including neuroimaging ( Panta *et al.*, 2016).

We propose a method to embed a decentralized dataset that is spread across multiple locations such that the data at each location cannot be shared with others into a 2D plane. We build the overall embedding by utilizing public, anonymized datasets. The method is similar to the landmark achievements previously used to improve computational efficiency ( De Silva & Tenenbaum, 2004; Silva & Tenenbaum, 2003). However, directly copying this approach does not produce accurate results, so we introduce a dynamic modification that generates an embedding that reflects relationships among points spread across multiple locations.

**Figure 1.**
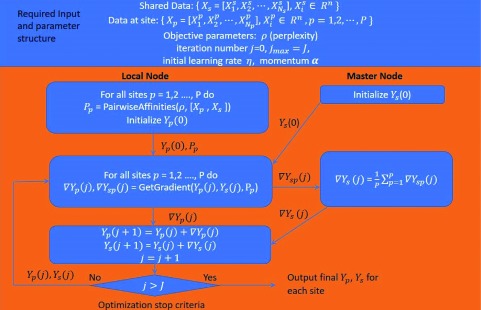
Multi-shot decentralized stochastic neighbor embedding (dSNE) algorithm.

The detailed algorithm diagram for decentralized multi-shot tSNE is demonstrated in Figure 1. *X _p_* and *X _s_* represent the high-dimensional site data and shared data, respectively. *Y _p_* and *Y _s_* represent the low-dimensional mapping site data and shared data, respectively. The master node initializes *Y _s_* and subsequently calculates a common gradient *∇Y _s_(j)* based on the site gradient *∇Y _sp_(j)* for each iteration *j* and update *Y _s_*, accordingly. Each local node will calculate the pairwise affinities among its own dataset and the shared dataset and then update *Y _p_* by locally calculating *∇Y _P_(j)*. With this scheme, *Y _s_* stays constant across all sites for every iteration and serves as a reference function. Meanwhile, *Y _s_* is influenced by *Y _p_*, which allows local embedding information to flow across the sites, resulting in a final map with less overlapping.

**Figure 2.**
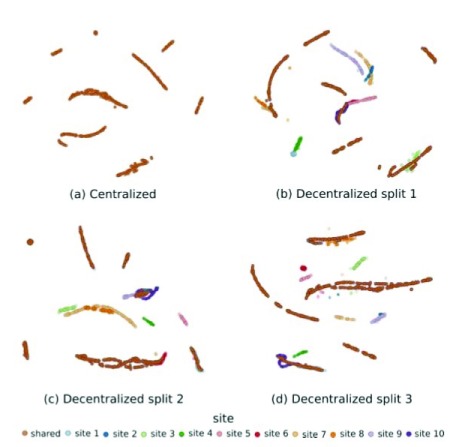
Decentralized stochastic neighbor embedding (dSNE) results for quality control metric of the ABIDE datasets ( Saha *et al.*, 2017). We randomly split the data into ten local and one reference dataset. The centralized results show ten different clusters. For three random splits of decentralized computation, we also obtain ten different clusters, and the number of clusters in the embedding is stable regardless of how the data are split among sites.

We have tested the performance of this algorithm by comparing the decentralized result with that of centralized tSNE using the quality control metric of the ABIDE dataset ( Di Martino *et al.*, 2014). The results demonstrate that the centralized and decentralized computations generate an equal number of clusters. Additionally, random splits do not affect the stability of the clusters ( Saha *et al.*, 2017). Please see Figure 2 for reference.

***Decentralized neural networks.*** Recently, deep learning has gained increasing attention because of its excellent performance in pattern recognition and classification, including in the neuroimaging domain ( Plis *et al.*, 2014). To enable both shallow and deep neural network computations within COINSTAC, we developed a feed-forward artificial neural network that is capable of learning from data distributed across many sites in a decentralized manner. We utilize mini-batch gradient descent to average the gradient across sites. For our purposes, each batch contains one sample per site. We then average the resulting gradients from the batch.

**Figure 3.**
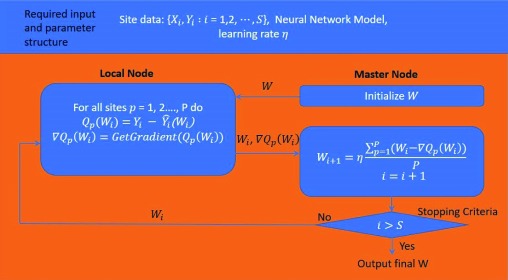
Decentralized neural network algorithm.

Figure 3 shows a flow chart of the decentralized neural network algorithm. As in a stochastic gradient descent (SGD) model, we calculate the error function *Q _p_*( *W _i_*) for each site *p* and *ith W. Q _p_*( *W _i_*). represent the discrepancy between the expected result *Y _i_* from the training set and the actual result from forward propagation ${\hat{Y}}_{i}(W_{i})$. Each site then sends *∇Q _p_*( *W _i_*) to the master node, which averages the gradient and returns the result to the sites. Each site then updates *W _i_* on the basis of the mini-batch gradient decent equation until all training data are exhausted. With the same initialization *W* in the master node, we find that *W _i_* is always shared across all sites, but the change in *W _i_* at each iteration is determined by the data at each site.

**Figure 4.**
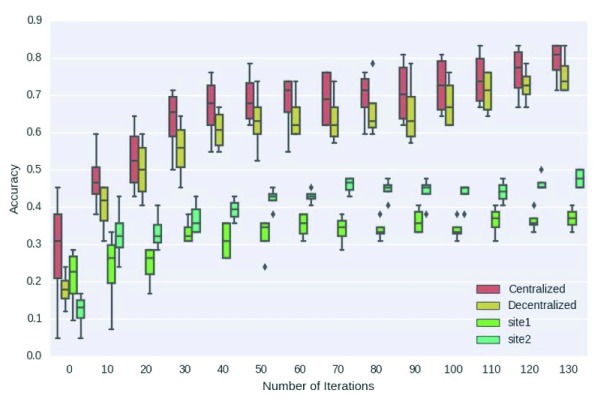
Experimental results for decentralized neural network using the Fagerström Test for Nicotine Dependence dataset addition functional MRI dataset ( Lewis *et al.*, 2017). In this experiment, we simulated an addiction dataset with two sites. The centralized classifier (red) and decentralized neural network classifier (yellow) perform similarly, and local sites classifiers (green and aquamarine) perform poorly.

We use a basic neural network known as a multilayer perceptron to demonstrate the decentralized computation process, but this framework can be easily extended to other types of neural networks. We tested the performance of this model using real functional magnetic resonance imaging (fMRI) data from smokers (Fagerström Test for Nicotine Dependence dataset) (Heatherton and Kozlowski 1992) and found that the decentralized model and pooled centralized model yielded similar classification accuracy, which vastly outperformed the accuracy at local, isolated sites ( Lewis *et al.*, 2017). Please see Figure 4 for reference.

***Decentralized joint ICA.*** When shared signal patterns are anticipated to exist among datasets, joint ICA (jICA) ( Calhoun *et al.*, 2006; Calhoun *et al.*, 2001; Sui *et al.*, 2009) presents a solution to combine and identify shared information over multiple datasets. Although originally proposed as a method for multimodal data fusion, jICA can also implement group temporal ICA of fMRI data. In both cases, datasets are concatenated (over modalities in multimodal fusion and over subjects across time in temporal ICA) and then jointly analyzed. The jICA model is particularly attractive for datasets where the number of observations is significantly smaller than the dimensionality of the data, as in temporal ICA of fMRI data (time points < voxels), as concatenation over datasets effectively increases the number of observations. In decentralized jICA (djICA), the datasets are stored at different sites, rendering the traditional centralized approach for concatenation ineffective. To solve this problem, we developed an implicit concatenation procedure based on the assumption that the data from each site will share the same global unmixing matrix.

**Figure 5.**
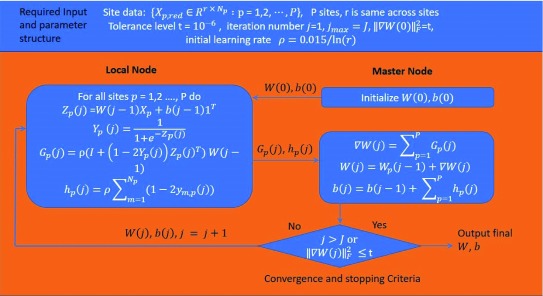
Decentralized joint independent component analysis (ICA) algorithm.

A diagram of djICA is shown in Figure 5. The global unmixing matrix includes *W* and bias *b*. Using this unmixing matrix, each site estimates the independent source *Z _p_*( *j*) and tries to maximize the entropy function of a sigmoid transformation of *Z _p_*( *j*) *(Y _p_*( *j*)). *G _p_*( *j*) and *h _p_*( *j*) are the local gradients for *W* and *b*, respectively. The master node sums the two gradients across all sites and updates the global unmixing matrix for the next iteration until either convergence or the stopping criteria is met.

**Figure 6.**
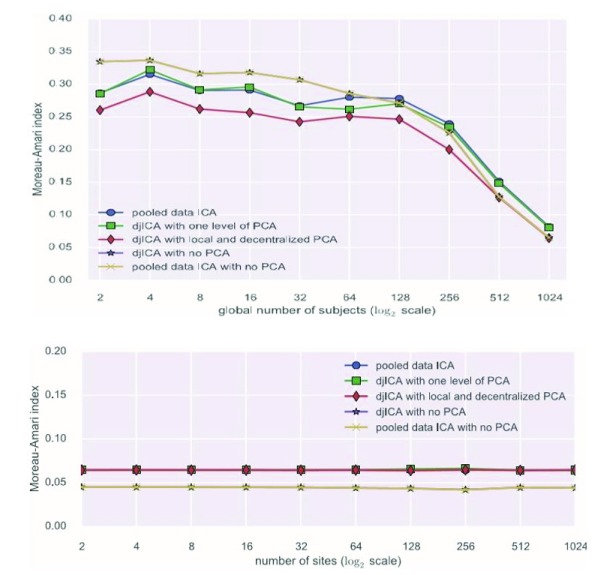
Experimental results for decentralized joint independent component analysis (djICA) ( Baker *et al*., 2015). The experiment is based on synthetic functional MRI data using a generalized autoregressive conditional heteroscedastic model ( Engle, 1982; Bollerslev, 1986). The top figure shows that as the global number of subjects increases, the Moreau-Amari index (MAI) decreases for both pooled-data ICA and djICA with different principal component analysis (PCA) operations. Additionally, MAI converges for pooled-data ICA and djICA when the number of subjects increases. The bottom figure shows that number of splits in the data have no effect on MAI.

The performance of djICA has been evaluated in studies by Plis *et al* ( Plis *et al.*, 2016) and Baker *et al* ( Baker *et al.*, 2015). The results of the experiments in these two studies convincingly demonstrate that with increased sample size the quality of feature estimation increases for both pooled-data ICA and djICA. Furthermore, we have found that splitting data across sites does not degrade the results given the same global data volume. Please see Figure 6 for reference.

***Decentralized IVA.*** When using joint ICA to decompose temporal or multimodal datasets containing a group of subjects, we make a strong assumption that the underlying source maps are identical across subjects. Clearly, it is more desirable for source maps to contain subject-specific features. IVA is an approach that allows corresponding sources from different subjects to be similar rather than identical. IVA enables the subject source maps to contain unique information, yet still be linked across different subjects ( Kim *et al.*, 2006; Silva *et al.*, 2016).

**Figure 7.**
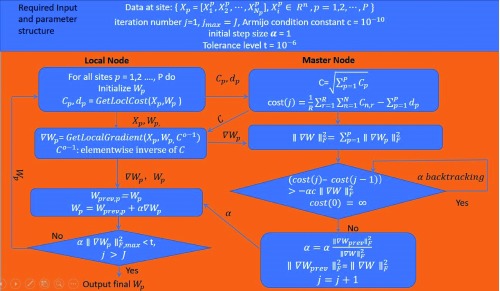
Decentralized independent vector analysis (IVA) algorithm.

We proposed a decentralized IVA (dIVA) method, which allows multiple institutions to not only collaborate on the same IVA problem but also spread the computational load to multiple sites, improving execution time. We use IVA with a Laplace assumption for the dependence structure of the underlying source groups ( Kim *et al.*, 2006; Lee *et al.*, 2008). Figure 7 shows a diagram of dIVA. Specifically, dIVA optimizes the same information measure as IVA by exploiting the structure of the objective function and fitting it into a decentralized computational model. In this model, a master node (or centralized aggregator) sends requests to local sites that contain the data. The sites send only data summaries ( *C _p_*, *d _p_*) back to the aggregator, which uses them to update a matrix of norms ( *C*) as well as the objective function (cost( *j*)). The aggregator sends this matrix back to the sites, which use its inverse ( *C* ^0–1^) to apply a relative gradient update on their local data. Subsequently, the local gradients are transmitted to the master node and aggregated to calculate a global step size ( *α*). *α* is then returned to the local sites to update their weights. This process is orchestrated iteratively by the local and master nodes until convergence, and results are stored at local sites.

**Figure 8.**
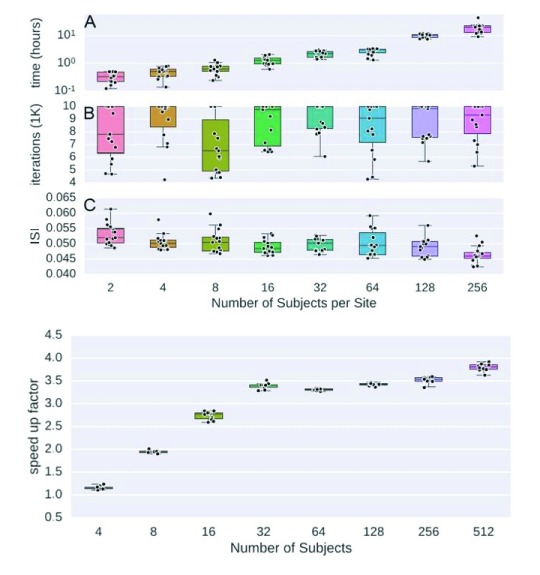
Experimental results for decentralized independent vector analysis (dIVA) ( Wojtalewicz *et al*., 2017). The experiment is based on synthetic data using a generalized autoregressive conditional heteroscedatic model and the SimTB functional MRI Simulation Toolbox ( Erhardt *et al*., 2012). The top figure shows how the processing time, number of iterations, and intersymbol interference (ISI) change as the global number of subjects increases. The processing time increases with the number of subjects per site ( **A**). Additionally, feature quality increases, indicated as decreasing ISI ( **C**). The bottom figure shows the processing time ratio between dIVA and IVA decreases as the global number of subjects increases. When the global number of subjects reaches 512, dIVA requires only one quarter of the processing time of IVA.

Figure 7 shows the optimization function utilized by IVA can be split across sites, allowing the bulk of the computation to be parallelized with the aid of an aggregator that collects summaries from individual sites. We have already evaluated our decentralized approach on synthetic sources, and experimental results show that dIVA provides high accuracy and significantly reduces the runtime of the method compared with a centralized computation ( Wojtalewicz *et al.*, 2017). Please see Figure 8 for reference.

### Improved COINSTAC user interface (UI)

**Figure 9.**
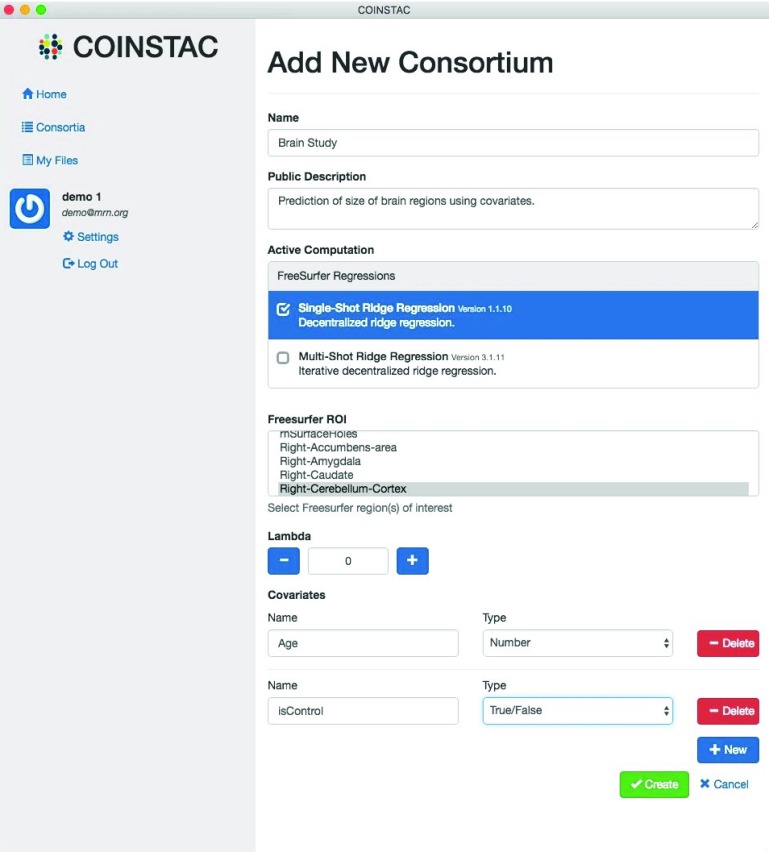
Example of how a consortium is created in the COINSTAC user interface.

We have improved the UI for COINSTAC by adding features that facilitate the input of brain imaging data, allow users to easily run computations, and keep users informed on the progress of the computation. To begin a collaborative, decentralized computation, a group of users that will participate in the analysis, called a *consortium*, must be created. This involves naming the consortium, choosing the computation, and defining the dependent and independent variables. The user who completes these steps is called the *consortium owner*. As shown in an example in Figure 9, the UI accepts FreeSurfer data saved in a comma-separated value (CSV) file as an input. The ROI of the brain computed by FreeSurfer is selected as the dependent variable in a ridge regression computation. Additionally, the regularization parameter (lambda), which limits overfitting in the model, is selected via a numeric field. A standard regression with no regularization is performed if lambda is given a value of zero.

**Figure 10.**
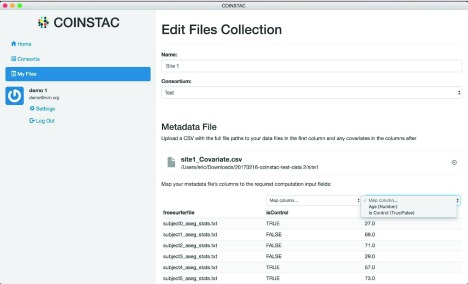
Example of binding files to a specific consortium in the COINSTAC user interface.

Next, the consortium owner declares the covariates (independent variables) and determines their types. The UI currently allows either Boolean (True/False) or numeric covariates. Every user who participates in the consortium must then choose a local data source, such as a FreeSurfer CSV file, and map the columns in the file to the variables declared by the consortium owner. Figure 10 shows how this is accomplished in the UI.

**Figure 11.**
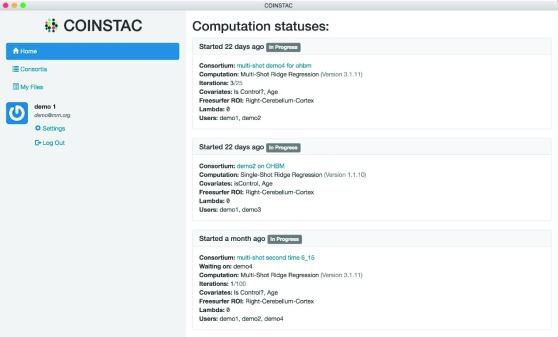
COINSTAC user interface computation status dashboard.

Once all the participants in the consortium have mapped columns in their local data sources to declared variables, the computation commences. The progress of computations in multiple consortiums is displayed on the Home tab of the UI. Figure 11 shows an example of this. In the top computation, a multi-shot ridge regression is on the third iteration out of a maximum of 25 iterations.

### New output statistics table with decentralized statistics computation for ridge regression

Regression analysis generates an equation to describe the statistical relationship between one or more predictor variables and the response variable. Decentralized ridge regression first produces the regression coefficients for all independent variables through an iterative optimization process. However, in most cases, a researcher may not only want to know the coefficient associated with certain regressor but also the statistical significance of this coefficient and the overall goodness of fit or coefficient of determination ( *R* ^2^) for the global model. In order to generate a standard statistical output accompanying the coefficient as in many major statistical tools, we developed a decentralized approach to calculate the t-values and goodness of fit for the global model without sharing any original data.

**Figure 12.**
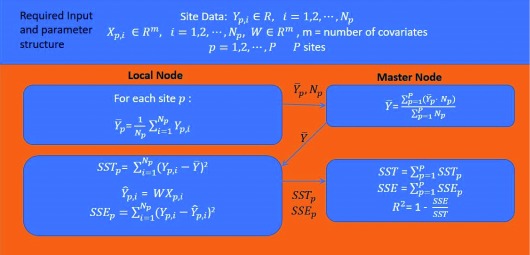
Decentralized *R* ^2^ calculation.

The decentralized *R* ^2^ calculation is demonstrated in Figure 12. First, each local node calculates the local average of dependent variable ${\bar{Y}}_{p}$ and transmits it and the size of dataset *N ^p^* to the master node. Then, the master node calculates the global $\bar{Y}$ and returns it to the local node. Subsequently, every node calculates the local total sum of squares ( *SST _p_*) and sum of squared errors ( *SSE _p_*) on the basis of $\bar{Y}$ and send them to the master node. Finally, the master node aggregates *SST _p_* and *SSE _p_* across all sites to calculate the global value of *R ^2^*.

**Figure 13.**
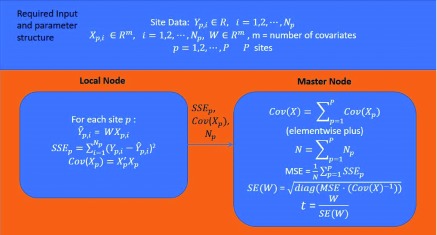
Decentralized t-value calculation.

The decentralized t-value calculation is demonstrated in Figure 13. Each local node calculates the local covariance matrix of *X _p_* and *SSE _p_* and transmits them and data size *N _p_* to the master node. The master node then aggregates *cov*( *X _p_*) to generate the covariance matrix of global covariates X to allow the following calculation of the t-values. MSE represents the mean squared error of the estimated coefficient W (or β).

After generating the t-value for every covariate and intercept, we use the public *distributions* library on npm ( https://www.npmjs.com/package/distributions) to generate the Student’s t-distribution and then calculate the two-tailed p-value for corresponding t-value.

**Figure 14.**
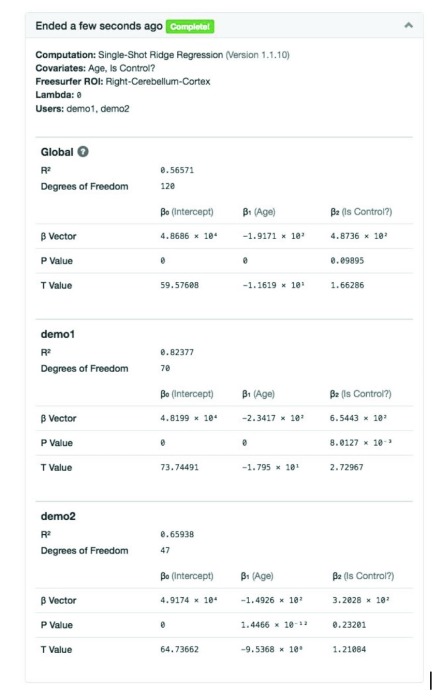
Example statistical output table for ridge regression. This output is generated using simulated freesurfer brain volume data. In the simulation, the intercept part (β _0_) was set to a fixed amount (48466.3 for Right-Cerebellum-Cortex); the age effect(β _1_) was selected randomly from range [-300, -100] and group(isControl) effect(β _2_) was selected randomly from range [500, 1000] for each pseudo subject; the standard unit Gaussian noise multiplied by random index ranged from 1800 to 2200 was added subsequently.

Figure 14 shows an example statistical output table for ridge regression. The COINSTAC UI displays the result with summarized consortium information at the top. In the output table, we first present the global fitting parameters, following by the fitting parameters locally calculated at each site. The COINSTAC UI also provides the detailed covariate name for each *β*.

### Complete pipeline specification

COINSTAC is not only designed to apply individual computations, but also to flexibly arrange multiple computations into a *pipeline*. Both decentralized analyses and local preprocessing steps can be included in a pipeline. The goal of COINSTAC is to provide a shared preprocessing script that is convenient for researchers and minimizes the data discrepancies across sites that become inputs to decentralized computations.

**Figure 15.**
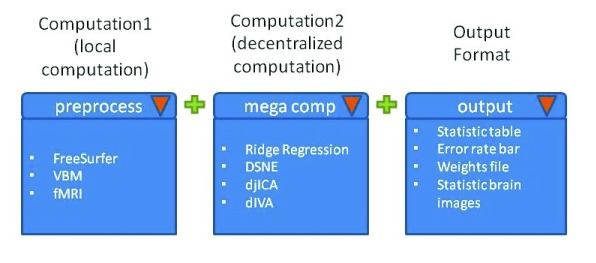
Example pipeline with one local, preprocessing computation and a decentralized computation. The output displayed in the user interface can be selected as well.

COINSTAC concatenates multiple computations into a pipeline and uses a *pipeline manager* to control the entire computation flow. Figure 15 shows a pipeline specification scheme with an initial preprocessing step and a following decentralized computation. Consortium owners will be able to select the computation step and output type through connected dropdown menus. After the computation steps have been selected, all users within a consortium will be shown cascading interfaces to upload input data and set hyperparameters for each computation. Additionally, the input from the latter computation step can be linked to the output from an earlier computation step.

**Figure 16.**
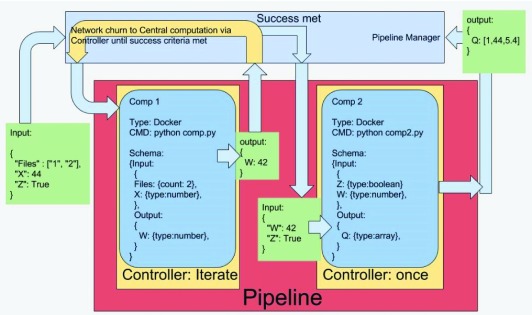
COINSTAC pipeline architecture. The pipeline manager handles the input and output of each pipeline, providing a conduit other nodes in the network. Each computation has its own schema that describes the names and types of its input and output parameters. Controllers are used to manage specific behavior in each computation in the pipeline. Each computation is encapsulated in a Docker container to improve portability among development environments.

Once a complete pipeline has been formed, all pipeline information is transmitted to the pipeline manager. Figure 16 shows how the pipeline manager interacts with a pipeline and its internal computations. The pipeline manager controls the entire computation flow. It is responsible for piping the input data to the first computation step, caching and transferring intermediate computation output, and storing the final pipeline output. An intermediate controller is added to provide fine-grained control for monitoring the iterative process between local and remote nodes for every computation. The computation *schema* is defined by a JavaScript object notation (JSON) structure and includes input and output specifications. A Docker container is used to encapsulate an individual computation block.

## Discussion

In this paper, we reviewed our progress on the development of decentralized algorithms that can be implemented on the COINSTAC platform. Every algorithm is structured similarly in that the local gradient of the objective function is transmitted to the master node, and the master node either returns a common averaged gradient or a step size (dIVA) to update the local weights. This scheme guarantees that information is shared across all sites on every iteration in the optimization algorithm to achieve a virtually pooled analysis effect (i.e., a mega-analysis). This framework also facilitates differential privacy by allowing for the addition of noise to each local objective function. We continue to develop decentralized algorithms as described below.

### Future decentralized algorithms

***Decentralized network gradient descent.*** SGD has emerged as the *de facto* approach to handle many optimization problems arising in machine learning, from learning classification/regression models to deep learning ( Bottou, 2010; Song *et al.*, 2013). For decentralized settings, SGD can be costly in terms of message complexity. We are currently developing approaches to limit this message complexity to enable a variety of statistical learning methods within COINSTAC. These approaches are guided by theory, but will involve developing task-specific heuristics to tune the algorithm parameters.

***Nonnegative matrix factorization (NMF).*** NMF is another popular method for discovering latent features in data such as images, where measurements are all nonnegative ( Lee & Seung, 2001). Although there has been significant work on NMF and its variants, the work on decentralized implementations is more limited, and the focus has been on improving parallelism for multicore systems ( Potluru *et al.*, 2014). Because of the message-passing nature of the COINSTAC architecture, we are developing decentralized and accelerated NMF algorithms that are optimized with gradient descent. Further extensions could allow users to find an NMF to minimize a variety of cost functions beyond squared error.

***Canonical correlation analysis (CCA).*** One challenging task in learning from multimodal or multiview data is to find representations that can handle correlations between the two views ( Sui *et al.*, 2012; Thompson, 2005). CCA is one such method. We are currently developing privacy-preserving CCA methods, as well as determining whether decentralized, message-passing approaches will be feasible within the COINSTAC architecture.

### Integration with large-scale collaborative frameworks

In recent years, the ENIGMA Consortium has conducted collaborative meta-analyses of schizophrenia ( van Erp *et al.*, 2016) and bipolar disorder ( Hibar *et al.*, 2017), in which subcortical brain volumes and cortical thicknesses were compared between patients and controls, respectively. In these studies, many univariate linear regression models were created in parallel to examine group differences for different regions of the brain. ENIGMA distributes analysis software to many sites and aggregates the results to conduct a meta-analysis. The upcoming version of COINSTAC will facilitate such studies by allowing researchers to specify models that contain combinations of selected dependent and independent variables. Table 1 elaborates on this point by showing an example in which a researcher selects a group of dependent variables (right and left cerebellum cortexes) and a group of independent variables (age and isControl). One model is computed separately for each combination of dependent and independent variables. The advantage of COINSTAC is that dissemination of software and aggregation of results will be handled by our software, eliminating many manual steps. In addition, as mentioned earlier, COINSTAC enables us to run multishot regression (hence converting a meta-analysis into a mega-analysis). Finally, COINSTAC opens up the possibility of running multivariate analysis (such as SVM ( Sarwate *et al.*, 2014) or IVA), as well as incorporating differentially private analyses, which would significantly extend the current ENIGMA approach, while also preserving the powerful decentralized model.

**Table 1.** Example parallel computation of combinations of two independent and two dependent variables.

Model	Independent variables	Dependent variables
1	Age	Right Cerebellum Cortex
2	isControl	Right Cerebellum Cortex
3	Age, isControl	Right Cerebellum Cortex
4	Age	Left Cerebellum Cortex
5	isControl	Left Cerebellum Cortex
6	Age, isControl	Left Cerebellum Cortex

## Software and data availability

COINSTAC is free and open source and can be downloaded at: https://github.com/MRN-code/coinstac

Archived source code as at time of publication: http://doi.org/10.5281/zenodo.840562 ( Reed *et al.*, 2017)

License: MIT

ABIDE dataset can be accessed at http://fcon_1000.projects.nitrc.org/indi/abide/

The Fagerström Test for Nicotine Dependence addiction dataset was collected within the Mind Research Network using local fMRI scanners. This dataset is stored in the Collaborative Informatics and Neuroimage Suit (COINS) https://coins.mrn.org/. This dataset is not a public dataset, but can be requested through COINS after receiving approval from the dataset owner.
