# Supplementary material for: COINSTAC: Decentralizing the future of brain imaging analysis
Source: F1000Res. 2017 Aug 18;6:1512. [Version 1] doi: 10.12688/f1000research.12353.1 (PMC5657031; doi:10.12688/f1000research.12353.1)
Supplement: Peer review history

# Peer review: Referee response for version 1

**DOI:** 10.5256/f1000research.13376.r26632

**Published:** 2017-10-25

**Authors:** Jens Foell

**Affiliations:** 1 Department of Psychology, Florida State University, Tallahassee, FL, USA

This manuscript discusses the development and use of the COINSTAC system, a framework that is meant to facilitate neuroimaging data sharing by allowing for easy decentralized analysis.

 The authors provide a thorough and easily understandable introduction into recent advances and challenges for neuroimaging data analysis: as technological barriers preventing the open sharing of large amounts of data have been removed, other obstacles have become apparent, ranging from common standards of analysis to legal issues.

 The manuscript demonstrates the use of the COINSTAC system with existing neuroimaging data to show that centralized and decentralized neural network classifiers lead to comparable results. The authors argue that one advantage of a decentralized analysis approach is that individual data cannot be easily compiled into a coherent data point, so that privacy is preserved through a process which essentially fragments and distributes individual data components. While this approach makes sense to me, I cannot judge whether this will in fact have an effect on the legal situation regarding the sharing of data between groups; this will likely depend on specifications given by regional jurisdictions or institutional bodies.

 Algorithms necessary for this decentralized processing are named and explained. Additional information includes specification of user interface and processing pipelines.

 Overall, this is a thorough and well-written article about software that is certainly needed to adapt to new challenges and opportunities pertaining to large-scale neuroimaging analyses and that will likely be useful to a large number of researchers.

 Minor comments:

- The term ‘mega-analysis’ is used without explanation, before being mentioned later on in the text with a quick description. I recommend defining the term at its first use, either in the text or as a footnote.
- For the different software packages mentioned, it should be made clear whether they are freely available or whether they need to be purchased. This could be mentioned in the main text, or the software can be included in the ‘Software and availability’ section at the end of the manuscript. One instance where this was missed is the mention of the Docker software.

I have read this submission. I believe that I have an appropriate level of expertise to confirm that it is of an acceptable scientific standard.

# Peer review: Referee response for version 1

**DOI:** 10.5256/f1000research.13376.r26634

**Published:** 2017-10-24

**Authors:** Joshua Balsters

**Affiliations:** 1 Department of Psychology, Royal Holloway, University of London, London, UK

This is a wonderful article describing an increasingly necessary resource. I have very little to add to this manuscript. 

1. The article mostly focuses on decentralized data analysis, however figure 15 highlights the preprocessing and output stages. It would be useful if the article included some information about the expected input formats. For example, does COINSTAC offer preprocessing tools? At the end of the first paragraph of the introduction the authors critique meta-analyses by suggesting "heterogeniety among studies caused by varying preprocessing methods applied to the data". Does COINSTAC offer tools to harmonize preprocessing, and if so what are they? Similarly, it would be good to have a summary figure of the output formats available. Can you visualise brain images online or do you have to download these?
2. Figure 8b is cropped

I look forward to seeing more additions and extensions to COINSTAC in the future.

I have read this submission. I believe that I have an appropriate level of expertise to confirm that it is of an acceptable scientific standard.
